# Audiometric evaluation after stapedotomy with Fisch titanium prosthesis

**DOI:** 10.5935/1808-8694.20130058

**Published:** 2015-10-04

**Authors:** Andre Luiz de Ataide, Gerson Linck Bichinho, Tatiana Mauad Patruni

**Affiliations:** a MSc. in Health Technology (Coordinator of the Cochlear Implant Group at the Pequeño Príncipe Hospital).; b PhD in Biomedical Engineering at the Université de Technologie de Compiègne (Professor in the Health Technology Graduate Program at PUC/PR).; c Specialist in Otorhinolaryngology (MD, ENT).

**Keywords:** ossicular replacement, otosclerosis, prostheses and implants, titanium

## Abstract

Otosclerosis causes the fixation of the stapes and conductive hearing loss, usually corrected with the use of hearing aids or through stapedotomy and the replacement of the involved stapes with a prosthesis. Titanium has been the most recently used material of choice in stapedotomy prostheses. Only two prostheses are commercially available in Brazil. There are no reports in the literature on the Fisch-type Storz titanium stapes piston prosthesis.

**Objective:**

This retrospective study aims to look into the auditory outcomes of patients submitted to stapedotomy and titanium stapes piston prosthesis implantation.

**Method:**

The criteria described by the American Academy of Otolaryngology were used to compare pre and postoperative air-bone gaps seen in audiometry tests.

**Results:**

The mean low-frequency postoperative air-bone gap was 12.9 dB; the mean high-frequency air-bone gap was 5.2 dB (mean 9.1 dB); median gap was 8.8 dB, with a minimum of 1.3 dB and a maximum of 21.6 dB; standard deviation was 5.7 dB, and *p* < 0.001. Twenty-five (75.8%) patients had air-bone gaps of 10 dB and under; 32 (96.9%) patients had gaps of 20 dB and under; and all patients had gaps of 30 dB and under.

**Conclusion:**

The Fisch-type titanium stapes piston prosthesis presented outcomes consistent with the literature and can be used safely in stapedotomy procedures.

## INTRODUCTION

Otosclerosis or otospongiosis is a hereditary disease characterized by degeneration of the otic capsule, focal bone neoformation, and increased local vascularization. The main clinical symptom described by patients is hearing loss, followed by tinnitus. This disease affects between 0,5% and 1,0% of the world's population, and presents bilateral involvement in 70% to 85% of the cases. Prevalence rates are higher among females and subjects in their thirties and forties^1^.

Prevalence varies based on ethnicity. Higher rates are seen among Caucasians, with up to 10% of such population presenting some degree of otosclerosis^2^.

The most frequently affected region of the optic capsule is the area around the oval window and the footplate of the stapes. The disease leads to the fixation of the stapes and consequently compromises the function of the ossicular chain, even when the malleus and the incus are normal. This is why conductive hearing loss is more common in otosclerosis, although mixed or sensorineural cases may also be observed, particularly in cases of extensive disease or cochlear otosclerosis. The malleus and incus are rarely involved^3^, ^4^.

Historically, the first description of stapes fixation to the oval window was based on an autopsy performed by Antonio Valsalva in 1753. The first stapes mobilization surgical correction was performed by Kessekl in 1878. Politzer and Sibenmann condemned the procedure in 1900 and it remained in disbelief until Rosen used it in 1953. But it was John Shea, in 1956, who introduced the stapedectomy procedure and performed the first stapedotomy in 1960.

The main goal in the treatment of otosclerosis is to improve patient hearing. This goal can be achieved by fitting patients with hearing aids or through otological microsurgery.

Despite the progressive improvements in the technological base and sound quality of hearing aids, surgery must be offered whenever possible as an option to improve hearing, given that most of the involved subjects are young adults, who often resist to the idea of wearing hearing aids, whether it is for cosmetic, social, or cultural reasons, and for whom surgery is a more pleasant and physiological means of recovering hearing^5^. Additionally, patients submitted to surgery are on average happier with their auditory outcome than subjects wearing hearing aids^6^.

Otological microsurgery, also known as stapedotomy or stapedectomy, has been the approach of choice of many surgeons and has been widely used in the treatment of otosclerosis^7^. The number of stapedotomy procedures varies significantly between countries, ethnic groups, and levels of access to health care^2^, ^6^, ^8^. The diseased stapes bone is removed during stapedotomy and is replaced with a piston prosthesis that conveys the sound stimulus from the incus to the footplate of the removed stapes.

Since the introduction of the concept of ossicular repair in 1950, many different materials have been used to manufacture prosthetic devices designed to repair the ossicular chain to its original anatomy and physiology and correct cases of conductive hearing loss. The search for the ideal prosthesis is an ongoing process. Autologous materials are often contaminated with prior infection and have limited availability. Homografts and tissue banks with ossicles from patients and cadavers have also been abandoned due to the risk of diseases being transmitted from donor tissue to the receptor^9^.

Since the introduction of Plastipore by Shea in 1976, ear surgeons have been waiting for a definitive solution in the area of alloplastic materials to provide them with biocompatibility, stiffness to convey sound, long-term duration, and minimal difficulty from the standpoint of the surgical technique and skill^9^. Many materials have been attempted, such as teflon, platinum, gold and titanium.

Titanium was first used in ossicular repair in Germany in 1993. Its advantages include significant tensile strength and low weight when compared to ceramics, plastics, and other metals. Titanium biocompatibility has also been alluded to by various authors^1^, ^10^.

Titanium prostheses have been correlated with excellent clinical outcome. Dalchow et al.^10^ published their extensive experience with more than 700 patients. Zenner (2001) et al. reported gains in frequencies between 2 and 3 KHz provided by the low weight and the stiffness of the titanium device.

In addition to the well-documented advantages in biocompatibility and function, titanium prostheses, unlike other metallic prostheses, do not pose hazard when exposed to the high intensity magnetic fields of MRI examination.

Only two makes of titanium stapes piston prostheses have been approved by the Brazilian Health Surveillance Authority (ANVISA). The device made by KURZ^®^ has been available for a few years in Brazil and its clinical outcomes and characteristics have been extensively described in the literature^4^, ^11^, ^12^.

The Fisch Titanium Piston made by Karl Storz in Germany was approved for use in Brazil on January 26, 2009. However, unlike most prostheses, the outcomes delivered by the Fisch Titanium Piston have not been described in the literature. This fact calls for an assessment on the characteristics, safety profile, and outcomes provided by this prosthesis.

### Objective

This retrospective study aimed to assess the pre and post-stapedotomy audiometry results of patients equipped with the Fisch Titanium Piston prosthesis.

• ***Specificobjectives***
  • To assess outcomes in four groups:
    - 10 dB and under
    - 20 dB and under
    - 30 dB and under
    - 30 dB and under
  • Verify gender incidences
  • Verify age distribution.

## METHOD

Thirty-three consecutive patients seen since January of 2009 were included in the study. Enrollment criteria were as follows:

• Patients with typical clinical history and complaint of hearing loss
• Unaltered tympanic membrane in the physical examination
• At least two preoperative audiometric tests suggestive of conductive hearing loss
• CT scans of the ear ruling out other less common anomalies that could mimic otosclerosis, such as fixation of the malleus, superior semicircular canal dehiscence syndrome, and enlarged vestibular aqueduct syndrome^13^
• Only the ears operated the first time were considered, never the previously operated contralateral ears
• Cases of primary surgery only.

Inadequate follow-up was considered as a reason to exclude subjects from the study. Patients with less than one year of postoperative follow-up were excluded.

The 33 cases included in the study were of first ears operated even in patients with bilateral involvement and of primary stapedotomy; cases of revision surgery were not included in the series.

This study is a retrospective analysis of the audiometric findings of patients submitted to stapedotomy for otosclerosis equipped with the Fisch Titanium Piston prosthesis made in Tübingen, Germany, listed in the manufacturer's catalog under number 277511.

The patients included in the study were aged between 23 and 64 years.

The study included consecutive patients operated since the ANVISA approval in January of 2009. All subjects were operated by the same surgeon at the same hospital using the endaural approach as described by Prof. Ugo Fisch. Their pre and postoperative audiometry test results were compared according to the criteria published by the Committee on Hearing and Equilibrium of the American Academy of Otolaryngology - Head and Neck Surgery Foundation. The Committee stated that postoperative outcomes may change with time and found that audiometry tests done one year or longer after surgery increase the reliability of clinical outcome interpretation^14^.

Pre and postoperative audiometry tests were performed for frequencies ranging between 0.5 KHz and 8 KHz for air conduction and 0.5 KHz to 4 KHz for bone conduction, including the frequency band of 3 KHz.

Auditory thresholds were calculated using the frequencies of 0.5, 1, 2, and 3 KHz. Air-bone gaps were calculated based on the difference between the mean air conduction thresholds and the mean bone conduction thresholds for the same frequencies. Preoperative air-bone gaps were compared to postoperative gaps observed one year or more after surgery to verify possible improvements in patient hearing^13^, ^14^.

Methodological variations often hamper comparisons between published audiometric findings after ossicular repair and stapedotomy. Some authors have used frequencies of 0.5, 1, and 2 KHz to calculate air-bone gaps, whereas others elected the 0.5, 1, 2, and 4 KHz bands. In 1995, the Committee on Hearing and Equilibrium of the American Academy of Otolaryngology recommended the use of the 0.5, 1, 2, and 3 KHz bands to include high frequencies relevant in speech recognition, thus reflecting ultimate goal of ossicular reconstruction^14^. The Committee also suggested that patients be assessed at least one year into follow-up^14^, ^15^, ^16^, although many authors have published short term results of stapedotomy and audiometric findings of patients being followed up for less than a year^13^.

The literature features studies carried out with 23 patients followed up for a mean of 25 weeks^17^, along with large reviews of 3,050 cases followed up for decades^7^. In order to standardize the audiometric findings after ossicular repair and stapedotomy, the Committee on Hearing and Equilibrium of the American Academy of Otolaryngology - Head and Neck Foundation published a set of guidelines in 1995^14^.

The value in decibels of the air-bone gap is defined by the difference between the preoperative and the postoperative gaps. A positive number is expected, thus reflecting the improvement in hearing after surgery. However, although uncommon, negative values may be observed in cases of poor surgical outcome.

Patients were divided into two groups in regards to how long after surgery they underwent audiometric testing: one group included subjects tested one year into follow-up (12 and 13 months) and another featured patients tested 13 months and longer after surgery.

A DF Vasconcelos MC 3103 microscope equipped with a lens with a focal distance of 250 mm was used in this study. Patient clinical data were obtained from chart management software Clinic^®^ developed by *Risc Informática*. Audiometry findings were acquired from Winaudio^®^, the hospital's audiometry data storage system.

The Committee recommends the calculation of standard deviations and indicates that results be shown in two modes: (1) postoperative air-bone gaps and (2) the closure of the air-bone gap in decibels or the gain in decibels of patients followed up for one year or longer, as results may change within the first months after surgery and become stabler after a year, thus allowing a more realistic analysis of outcome.

Another frequently used form of presenting results is by categorizing outcomes into four groups: subjects with postoperative air-bone gaps of 10 dB and under (reflecting excellent surgical outcome); patients with gaps of 20 dB and under; individuals with gaps of 30 dB and under; and cases with gaps greater than 30 dB (poor outcome stapedotomy)^14^.

Therefore, the values in decibels of the air-bone gap closure after surgery were determined by the difference between the preoperative and postoperative air-bone gaps at 0.5, 1, 2, and 3 KHz.

This study was approved by the National Research Ethics Committee (CONEP) and granted permit FR-469647.

## RESULTS

Data collection was carried out in november and december of 2011. Analysis was performed based on comparisons between pre and postoperative values, lower and higher frequencies, age, gender, and time between surgery and audiometric testing.

### Statistical analysis

The results observed in this study were described in the form of mean values, medians, minimum and maximum values, standard deviations (quantitative variables) or frequencies and percentages (qualitative variables). The Wilcoxon signed-rank test was used to compare pre and postoperative audiometric test values. The Mann-Whitney U test was used to compare groups in terms of audiometry variables. Statistical significance was attributed to *p* < 0.05. Statistical analysis was performed using software program Statistica v.8.0^®^.

Twenty-nine of the 33 subjects had 4.5 mm prostheses implanted and four had 4.75 mm prostheses put in place. Half a millimeter was added in relation to the original measurement while the prostheses were being processed on the cutting block, to account for the part of the prosthesis that was inserted through the footplate.

### Pre and postoperative air-bone gaps

The null hypothesis in which results were equal before and after surgery was tested versus the hypothesis in which results were different, considering each of the variables defined for air-bone gaps (mean values in all frequencies, mean value in low frequencies, and mean value in high frequencies). Table 1 shows the descriptive statistics related to air-bone gaps and the *p*-values arising from statistical analysis.

**Table 1:** Air-bone gaps in each frequency range.

Variable	Test	n	Mean	Median	Minimum	Maximum	Standard deviation	p-value*
Mean air-bone gap - all frequencies	Before surgery	33	28.1	27.5	20.0	36.3	4.8	< 0.001
	After surgery	33	9.1	8.8	1.3	21.3	5.7	

Mean air-bone gap - low frequencies	Before surgery	33	35.6	35.0	22.5	47.5	7.4	< 0.001
	After surgery	33	12.9	12.5	0.0	30.0	8.1	

Mean air-bone gap - high frequencies	Before surgery	33	20.5	20.0	15.0	30.0	4.5	< 0.001
	After surgery	33	5.2	2.5	0.0	12.5	3.9	

* Wilcoxon signed-rank test, *p* < 0.05. Source: author, 2012.

**Graph 1:**
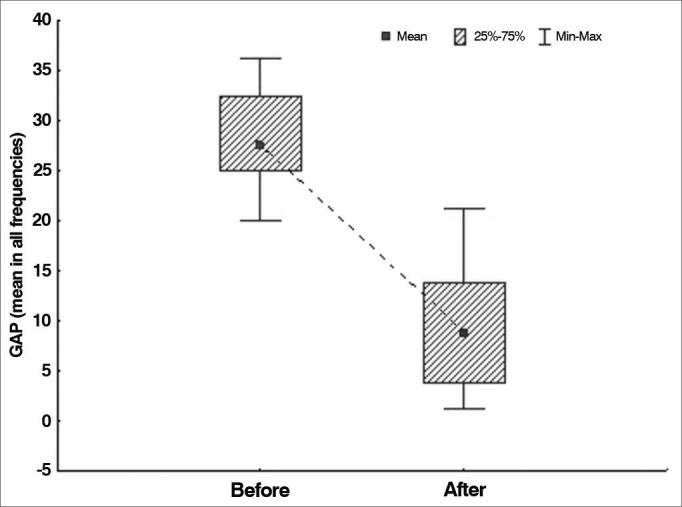
Air-bone gap distribution in all frequencies before and after surgery.

**Graph 2:**
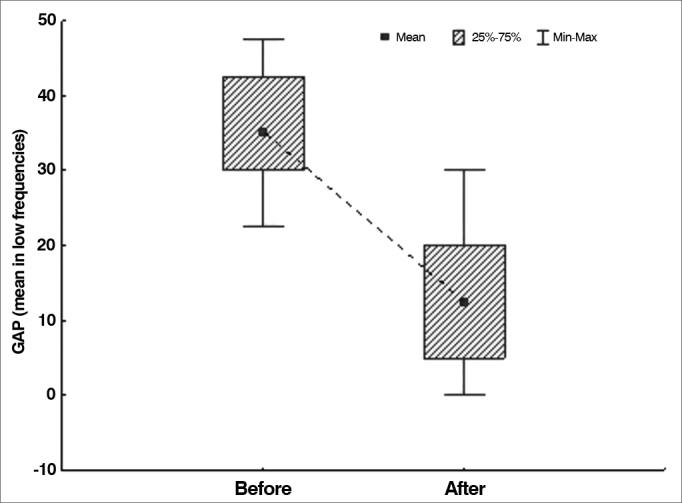
Air-bone gap distribution in low frequencies before and after surgery.

**Graph 3:**
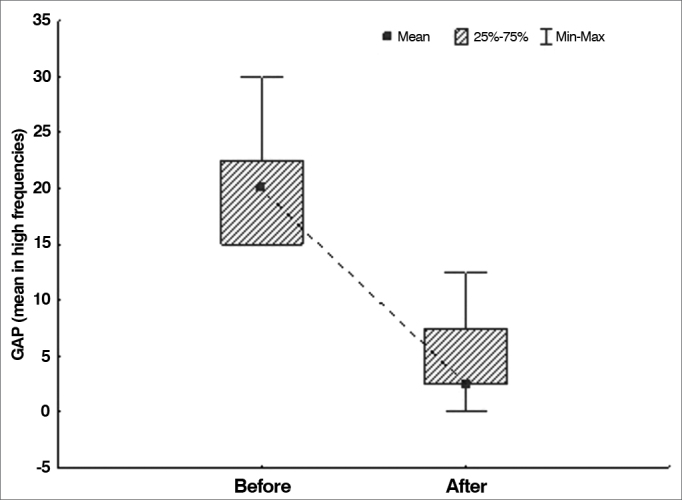
Air-bone gap distribution in high frequencies before and after surgery.

The Graph 1, Graph 2, Graph 3 illustrate the findings.

### Frequencies and percentages for each gap range

Table 2 describes the distribution of cases by postoperative air-bone gap range. Groups are cumulative, i.e., the group with gaps of 10 dB and under is included in the group with gaps of 20 dB and under, and both are included in the group with gaps of 30 dB and under.

**Table 2:** Patients in each air-bone gap interval.

Gap	Cases (after surgery)
≤ 10	25 (75.8%)
≤ 20	32 (96.9%)
≤ 30	33 (100%)
> 30	0 (0%)

### Gain after surgery

**Graph 4:**
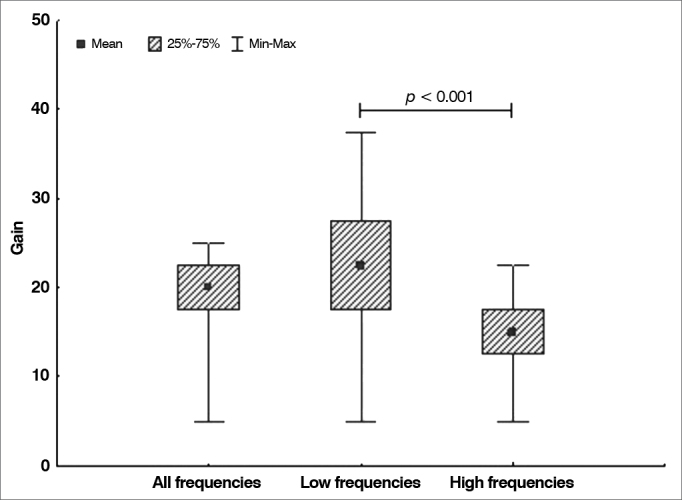
Distribution of hearing gain in decibels according to frequency.

The null hypothesis in which gains at low frequencies were equal to the gains at high frequencies was tested against the hypothesis in which the results were different. Presents the descriptive statistics for gain considering all frequencies, low frequencies, and high frequencies alone. The *p*-value arising from the comparison of low and high frequencies is also shown. Table 3 and Graph 4 illustrate the data.

**Table 3:** Hearing gain in decibels for each frequency range.

Variable	n	Mean	Median	Minimum	Maximum	Standard deviation	*p*-value*
Gain in all frequencies	33	19.0	20.0	5.0	25.0	5.3	-

Gain in low frequencies	33	22.7	22.5	5.0	37.5	8.1	<0.001
Gain in high frequencies	33	15.3	15.0	5.0	22.5	4.5	

* Wilcoxon signed-rank test, *p* < 0.05. Source: author, 2012.

### The effect of age upon hearing gain

**Graph 5:**
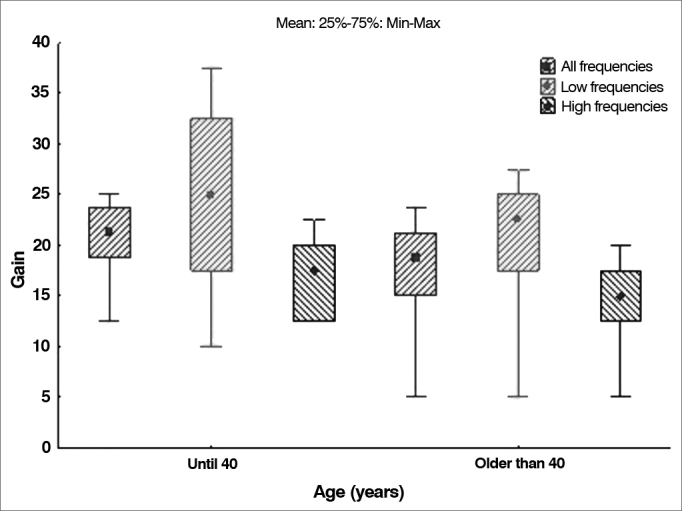
Distribution of hearing gain according to frequency for each age range.

The mean age of the patients enrolled in the study was used to divide them into two groups: subjects aged 40 and under, and subjects older than 40. The null hypothesis in which results were equal was tested against the hypothesis in which they were different for all variables related to gain (all frequencies, low frequencies, and high frequencies). Presents the descriptive statistics related to gain for each group, along with the *p*-values derived from statistical calculations. Table 4 and Graph 5 illustrate the distribution.

**Table 4:** Hearing gain in decibels in different patients age groups.

Variable	Age group	n	Mean	Median	Minimum	Maximum	Standard deviation	*p*-value*
Gain in all frequencies	40 and younger	19	20.8	21.3	12.5	25.0	3.5	0.042
	Over 40	14	16.6	18.8	5.0	23.8	6.5	

Gain in low frequencies	40 and younger	19	25,0	25.0	10.0	37.5	7.5	0.114
	Over 40	14	19.6	22.5	5.0	27.5	8.0	

Gain in high frequencies	40 and younger	19	16.6	17.5	12.5	22.5	3.4	0.163
	Over 40	14	13.6	15.0	5.0	20.0	5.3	

* Wilcoxon signed-rank test, *p* < 0.05. Source: author, 2012.

### The effect of gender upon hearing gain

**Graph 6:**
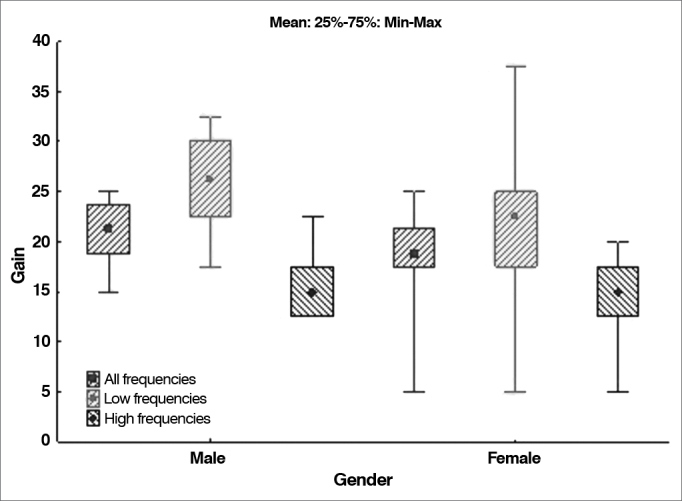
Distribution of hearing gain by gender.

The null hypothesis in which gain results were the same for males and females was tested against the hypothesis in which they were different, considering each of the variables related to gain (all frequencies, low frequencies, and high frequencies). Presents the descriptive statistics related to gain for each gender, along with the *p*-values derived from statistical calculations. Table 5 and Graph 6 illustrate the data.

**Table 5:** Hearing gain distribution by gender.

Variable	Gender	n	Mean	Median	Minimum	Maximum	Standard deviation	*p*-value*
Gain in all frequencies	Female	21	17.9	18.8	5.0	25.0	6.0	0.141
	Male	12	21.0	21.3	15.0	25.0	3.2	

Gain in low frequencies	Female	21	20.7	22.5	5.0	37.5	8.9	0.048
	Male	12	26.3	26.3	17.5	32.5	4.7	

Gain in high frequencies	Female	21	15.0	15.0	5.0	20.0	4.9	0.897
	Male	12	15.8	15.0	12.5	22.5	3.7	

* Wilcoxon signed-rank test, *p* < 0.05. Source: author, 2012.

### The effect of time between preoperative and postoperative testing upon gain

**Graph 7:**
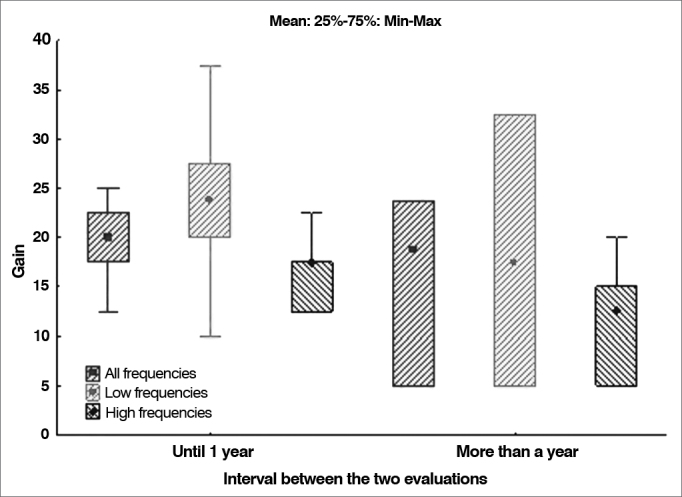
Distribution of hearing gain by time to test after surgery.

Patients were divided into two groups: subjects tested one year after surgery and individuals tested more than a year after surgery. The first group included patients with audiometry tests done 12 to 13 months apart, and the second featured subjects tested 13 months and longer after surgery. The null hypothesis in which gain results were the same for both groups was tested against the hypothesis in which they were different, considering each of the variables related to gain (all frequencies, low frequencies, and high frequencies). Presents the descriptive statistics related to gain for each group, along with the *p*-values derived from statistical calculations. Tables 6 and 7 and Graph 7 illustrate the data.

**Table 6:** Distribution of hearing gain by time to test after surgery.

Variable	Time interval between tests	n	Mean	Median	Minimum	Maximum	Standard deviation	*p*-value*
Gain in all frequencies	One year	7	15.0	18.8	5.0	23.8	9.1	0.424
	Over a year	26	20.1	20.0	12.5	25.0	3.2	

Gain in low frequencies	One year	7	18.9	17.5	5.0	32.5	13.4	0.476
	Over a year	26	23.8	23.8	10.0	37.5	5.9	

Gain in high frequencies	One year	7	11.1	12.5	5.0	20.0	6.1	0.039
	Over a year	26	16.4	17.5	12.5	22.5	3.2	

* Wilcoxon signed-rank test, *p* < 0.05. Source: author, 2012.

**Table 7:** Mean and median age and length of postoperative follow-up.

Variable	n	Mean	Median	Minimum	Maximum	Standard deviation
Age (years)	33	40.5	40.0	24.0	52.0	7.6
Postoperative follow-up (years)	33	1.55	1.34	0.12	3.25	0.81

### Distribution by gender and ear side

Tables 8 and 9 show the distribution of patients by gender and side of the operation, respectively.

**Table 8:** Distribution by gender.

Gender	n	Percentage
Male	12	36.4
Female	21	63.6
Total	33	100.0

**Table 9:** Distribution by side.

Side	n	Percentage
Left	12	36.4
Right	21	63.6
Total	33	100.0

## DISCUSSION

Otosclerosis is a relatively common occurrence in the practice of otorhinolaryngology and audiology, as it is seen in 10% of the caucasian population^2^ and less frequently in other ethnic groups^8^. The diagnosis is eminently clinical and audiological, although some authors have described the usefulness of CT imaging to confirm diagnoses and assess the extent of involvement^2^. In this study, CT scans were ordered for all patients before surgery.

Piston prosthesis of various designs made from one material, alloy compounds, and even two different materials have been used with advantages and disadvantages^7^, ^11^, ^12^.

Many authors have described the superiority of titanium in relation to other materials. The KURZ^®^ device faced distribution issues in Brazil for some time, but the ANVISA approved the prosthesis used in this study in january of 2009.

Unlike the STORZ^®^ prosthesis, the KURZ^®^ device has been cited in numerous publications^4^, ^7^, ^11^, ^12^ and described in a study done exclusively on it*.* The lack of published data on the results provided by the Fisch Titanium Piston prosthesis and the increase in its use in Brazil called for a specific study.

This study looked into 33 consecutive patients implanted with Fisch Titanium Piston prostheses*.* The subjects had been diagnosed with otosclerosis and were analyzed based on the surgery done on the first operated side. All cases were of primary surgery, no cases of revision surgery were included. Results were analyzed retrospectively at frequencies of 0.5, 1, 2, and 3 KHz, as per the guidelines published by the Committee on Hearing and Equilibrium of the American Academy of Otolaryngology^14^.

The mean postoperative air-bone gap of the patients included in this study for the frequencies mentioned above was 9.1 dB; the median was 8.8 dB; the minimum *gap* was 1.3 dB; the maximum gap was 21.6 dB; standard deviation was 5.7 dB; and *p* < 0.001.

When low frequencies were considered, the mean gap was 12.9 dB. High frequencies yielded a mean gap of 5.2 dB, indicating that the titanium prosthesis could effectively restore ossicular chain function.

The division of postoperative auditory results into 10 dB intervals as recommended by the guidelines of the American Academy of Otolaryngology revealed that 25 (75.8%) patients had air-bone gaps of 10 dB and under; 32 (96.9%) had gaps of 20 dB and under; and all cases had air-bone gaps of 30 dB and under.

The Committee on Hearing and Equilibrium considers that postoperative air-bone gaps of 10 dB and under reflect excellent surgical outcome^14^. However, other authors consider that good outcome has been achieved with postoperative gaps of 20 dB and under^11^, ^17^. If this criterion had been used in this study, our surgery success rate would have been of 96.9%.

These outcomes may be comparable to those of another titanium prosthesis used in Brazil, as reported in a paper published in 2003 by a group from the Netherlands, in which 79% of the patients had air-bone gaps of 10 dB and under and 98% of the subjects had gaps of 20 dB and under. However, it should be pointed out that the outcomes in the Dutch study were based on short-term postoperative audiometry, i.e., the tests were carried out within less than a year of surgery, against the guidelines published by the Committee on Hearing and Equilibrium from the American Academy of Otorhinolaryngology.

Vicent et al.^7^ reported 93% of patients with gaps of 10 dB and under using a teflon prosthesis. Tange et al.^11^ published a study comparing gold and titanium prostheses. The two performed satisfactorily, but the titanium device yielded 94% of good results with a mean postoperative air-bone gap of 7.6 dB, against 91% of the gold prosthesis with a mean gap of 11.6 dB.

In 2010, Manghan compared the outcomes of platinum and nitinol (nickel and titanium alloy) prostheses based on the same criteria used in our study, and reported a rate of success of 96% with the platinum device and 92% with the nitinol prosthesis^18^. In 2009, Fayad analyzed a series of 416 procedures and found inferior outcomes, with 78.3% of the patients presenting air-bone gaps of 10 dB and under with the nitinol prosthesis, also known as Smart Piston Prosthesis^16^. In 2011, Ying et al.^19^ looked into 190 cases of stapedotomy implanted with the nitinol prosthesis and described good short term outcomes, but a rate of late complications above 11%, mostly due to lateral shifts of the prosthesis resulting from detachment from the long process of the incus. This adverse event is much more commonly seen in prostheses made with other materials, and significantly deteriorates the quality of long term outcomes.

**Chart 1:**
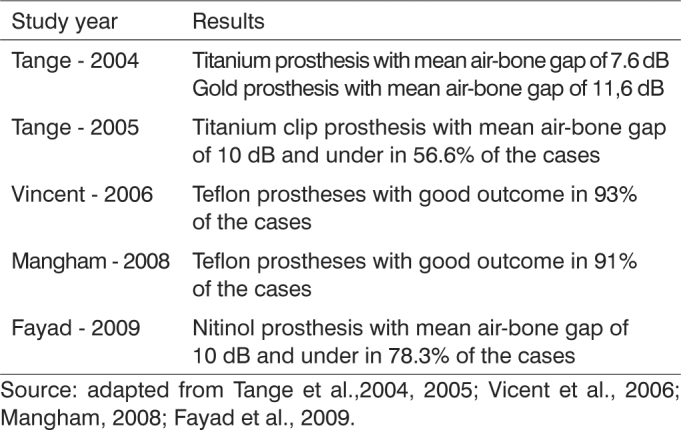
Results of studies on prostheses.

Source: adapted from Tange et al.,2004, 2005; Vicent et al., 2006; Mangham, 2008; Fayad et al., 2009.

The experience with titanium clip prostheses published by Grolman & Tange^17^ revealed less consistent results, with air-bone gaps of 10 dB and under being observed in only 56.6% of the cases. In another study, Mangham^20^ compared the same titanium clip prosthesis to teflon devices and reported worse results with the titanium clip prostheses (91% vs. 84% success rate). Chart 1 describes the outcomes of the main papers published within the last eight years on the matter.

This study found significant improvement in air conduction at high frequencies. Previous studies have also described greater gains in higher frequencies in patients wearing teflon prostheses, also light weight devices as the ones made with titanium, when compared to heavier prostheses such as the ones made with gold^4^, ^11^.

In terms of age, this study found that the mean hearing gains in all frequencies were statistically higher in patients aged 40 and under. The literature contains similar reports, with more significant improvements seen in younger populations. In our series, the youngest patient was 29 and the oldest 65. Although rare, children (subjects aged 18 and under) may also be affected by otosclerosis. Vincent analyzed 3,050 stapedotomy procedures carried out during a period of 15 years, and found 34 had been performed in children. However, reports such as this involving patients under 18 and subjects aged 65 and older submitted to stapedotomy are very rare^7^.

The literature indicates that incidence rates are significantly higher in female subjects^21^, ^22^, ^23^, ^24^. Reported ratios range from 2:1 to 3:1 in some series^1^, ^21^, ^22^, ^23^. Hormonal differences have been mentioned as a possible explanation for the higher prevalence in females, and reports of worsening condition after pregnancy have been described in the literature. But this issue is yet to be fully comprehended^22^, ^23^. In our study, 21 of the 33 patients were females, thus keeping with the 2:1 ratio previously described for individuals with otosclerosis.

The time after surgery one should wait before testing the patient's hearing is a very important factor in the assessment of auditory outcomes^6^, ^21^, as good results seen early on within months of surgery may deteriorate during long term follow-up^9^, ^23^. In light of this fact, the Committee on Hearing and Equilibrium of the American Academy of Otolaryngology recommended that audiological tests should be carried out at least a year after surgery^14^.

Although some papers have been published without following the guidelines^17^, the vast majority of the authors have chosen to comply with them^7^, ^11^, ^12^, ^25^. Audiometric tests done within less than a year after surgery were not included in this study. The hypothesis that audiometry tests done within exactly a year would yield better results than tests done within over a year of surgery was tested. Although the literature indicates that the results of longer term follow-up of more than a year tend to deteriorate^7^, ^12^, our series suggested that audiometry tests done over a year after surgery had slightly better results than the tests performed exactly within one year of surgery. This finding may be explained by the small number of cases tested within exactly a year (7), against the 26 subjects submitted to audiometry tests over a year after surgery.

Many authors have elected crimping as the most difficult step in the stapedotomy procedure^17^. The literature describes three alternatives to this step. The most common is using teflon prostheses. Given the memory present in this material, the loop is enlarged by the surgeon, placed in the incus, only to return to its original shape after a few seconds^7^. The second alternative is the nitinol prosthesis, which crimps with heat. However, heat can damage the incus and introduce late erosion of the incus by ischemia. Allergy to nickel may also be a factor in nitinol prostheses^26^, ^27^. The third is the titanium clip prosthesis, which is fitted onto the incus, but its results are not as good as those observed for other devices^18^.

However, in these three alternatives the surgeon has no control over how tight the prosthesis is in the incus, which may leave it either too tight or too loose and lead to problems related to inadequate device stability, thus increasing the risk of poor fitting in incus bones of extreme sizes and lateral migration of the prosthesis^4^, ^21^, ^23^. Therefore, the literature shows that despite the self-fitting alternatives, manual crimping is still a safe, effective procedure. The Fisch Titanium Piston prosthesis requires manual crimping, and no cases of lateral migration of the prosthesis or erosion of the long process of the incus were observed in our series.

The most common piston prosthesis diameters described in the literature are 0.4 and 0.6 mm^12^. The Fisch Titanium Piston prosthesis is available only in the 0.4 mm diameter, unlike other devices which are usually available in 0.6 mm^4^, ^7^, ^12^, ^26^. Smaller diameters mean smaller platinotomy incisions, and possibly lower risk of inner ear injury and hearing loss, and fewer postoperative symptoms of vertigo. However, specific studies on the impact of prosthesis diameter on auditory outcome failed to show such advantages^12^. In this study, none of the patients had prolonged incapacitating vertigo or signs of hearing loss after surgery.

Scanning electron microscopy of the surface of the prostheses indicates that gold devices are too rough, teflon prostheses are too smooth, and titanium devices present an intermediate level of roughness^4^, ^26^. Titanium devices are also light, as are teflon prostheses, with weights close to the human stapes, of about 2.8 mg, unlike gold, which is four times heavier. This improves outcome in high frequencies and discrimination^11^. Additionally, it is a solid piece, without connections as the teflon/ platinum and teflon/steel devices, which may separate in the long term^18^.

Despite the discussion on materials, shapes, and approaches, the literature has unanimously determined that surgeon experience is a decisive factor that accounts for more than 80%^26^ of the success of stapedotomy procedures.

## CONCLUSION

Despite the small size of the sample, the audiometric results reported in this study suggested that the outcomes provided by the Fisch Titanium Piston prosthesis were comparable to those of other prostheses described in the literature. It can be used safely in otosclerosis surgery and serves as a good option for stapedotomy procedures, particularly when the surgeon prefers to use titanium devices. The study indicated that hearing gains were larger in patients under 40 years of age. Incidence rates of otosclerosis were higher in females, as previously described in the literature, but no gender differences were seen in hearing gain.
